# Quercitrin Is a Novel Inhibitor of *Salmonella enterica* Serovar Typhimurium Type III Secretion System

**DOI:** 10.3390/molecules28145455

**Published:** 2023-07-17

**Authors:** Qingjie Li, Lianping Wang, Jingwen Xu, Shuang Liu, Zeyu Song, Tingting Chen, Xuming Deng, Jianfeng Wang, Qianghua Lv

**Affiliations:** 1 Research Center of Traditional Chinese Medicine, The Affiliated Hospital to Changchun University of Chinses Medicine, Changchun 130021, China; lqj19811005@163.com; 2 School of Traditional Chinese Medicine, Jilin Agricultural Science and Technology University, Changchun 132101, China; ziliaowlp@163.com; 3 State Key Laboratory for Diagnosis and Treatment of Severe Zoonotic Infectious Diseases, Key Laboratory for Zoonosis Research of the Ministry of Education, Institute of Zoonosis, College of Veterinary Medicine, Jilin University, Changchun 130062, China; xu18626969503@163.com (J.X.); 15504421028@163.com (Z.S.); chentt20@mails.jlu.edu.cn (T.C.); dengxm@jlu.edu.cn (X.D.); 4 Jilin Jinziyuan Biotech Inc., Shuangliao 136400, China; jzyliushuang@126.com; 5 Institute of Animal Science and Veterinary Medicine, Shandong Academy of Agricultural Sciences, Jinan 250100, China

**Keywords:** *Salmonella enterica* serovar Typhimurium, type III secretion system, T3SS inhibitor, anti-virulence, quercitrin

## Abstract

The purpose was to screen type III secretory system (T3SS) inhibitors of *Salmonella enterica* serovar Typhimurium (*S. Typhimurium*) from natural compounds. The pharmacological activities and action mechanisms of candidate compounds in vivo and in vitro were systematically studied and analyzed. Using a SipA-β-lactamase fusion reporting system, we found that quercitrin significantly blocked the translocation of SipA into eukaryotic host cells without affecting the growth of bacteria. Adhesion and invasion assay showed that quercitrin inhibited *S. Typhimurium* invasion into host cells and reduced *S. Typhimurium* mediated host cell damage. β-galactosidase activity detection and Western blot analysis showed that quercitrin significantly inhibited the expression of SPI-1 genes (*hilA* and *sopA*) and effectors (SipA and SipC). The results of animal experiments showed that quercitrin significantly reduced colony colonization and alleviated the cecum pathological injury of the infected mice. Small molecule inhibitor quercitrin directly inhibited the function of T3SS and provided a potential antibiotic alternative against *S. Typhimurium* infection. Importance: T3SS plays a crucial role in the bacterial invasion and pathogenesis of *S. Typhimurium*. Compared with conventional antibiotics, small molecules could inhibit the virulence factors represented by *S. Typhimurium* T3SS. They have less pressure on bacterial vitality and a lower probability of producing drug resistance. Our results provide strong evidence for the development of novel inhibitors against *S. Typhimurium* infection.

## 1. Introduction

*Salmonella* is a Gram-negative enterobacteriaceae commonly found in the external environment, which also exists in the intestinal tract of humans and animals [1]. *Salmonella* has a wide range of hosts, primarily causing disease in animals such as chickens, pigs, horses, and cattle. In addition, it causes contamination of animal food. It mainly causes human diseases such as typhoid, paratyphoid, and gastroenteritis through the fecal–oral pathway [2]. *Salmonellosis* is one of the most common food-borne diseases in humans [3]. Typhoid fever caused by *S. Typhimurium* remains a significant human health problem, especially in developing countries [3]. In short, *Salmonella* hinders the development of the livestock industry, endangers food safety, and causes panic about public health safety issues.

Antibiotics are still the first choice to treat *Salmonellosis*. However, antibiotic resistance is leading to a huge challenge in preventing the spread of infectious diseases caused by *Salmonella* [4]. New anti-infection therapies are urgently needed, such as using alternative drugs to prevent or treat pathogen infections. *S. Typhimurium* induces inflammatory diarrhea and invades non-phagocytic epithelial cells using the type III secretion system (T3SS) encoded by SPI-1 [5], which is critical to bacterial infection. T3SS is a needle-like structure that injects several effectors into the eukaryotic host cytoplasm [6]. Structural genes for the functional T3SS device assembling and effectors are encoded in the SPI-1 *prg*/*org*, *inv*/*spa*, and *sic*/*sip* operons; meanwhile, three AraC-like regulators, *hilD*, *hilC*, and *rtsA*, control the expression of *hilA* in SPI-1 signal cascade pathway activation [7]. HilD is the dominant regulator, while HilC and RtsA act as signal amplifiers [8]. SipA can bind to host cell actin and promote bacterial internalization [9]. The killing effect of macrophages is caused by *Salmonella*-induced apoptosis and occurs after caspase-1 is activated by SipB [10]. SipC is responsible for the translocation of effectors and the regulation of actin [11]. Thus, T3SS has become an attractive drug screening target to reduce the risk of antimicrobial resistance by indirect sterilization or antimicrobial dependence when treating bacterial infection. Today, there are multiple natural compounds, such as Tannic Acid, Thymol, and Fraxetin, that can target the type III secretion system of *Salmonella* to resist *Salmonella* infection. Therefore, it is a new strategy to screen inhibitors of the type III secretion system to resist *Salmonella* infection by using natural compounds that do not easily develop drug resistance as found in the inhibitor screening library.

Through screening in the library containing 354 compounds, it was found that a glycosylated flavonoid compound named quercitrin, mainly isolated from the whole grass of Hypericum perforatum, could significantly inhibit the biological function of *Salmonella* T3SS, rather than the direct antibacterial or bactericidal mechanism. In addition, the pharmacological activity of quercitrin in vitro was evaluated by eukaryotic cell adhesion, invasion, and gentamicin protection assay, and its preliminary protective effect was confirmed in vivo. In conclusion, quercitrin could be used as a potential drug for the prevention and treatment of *Salmonella* infections.

## 2. Results

### 2.1. Screening Natural Compound Inhibitors of S. Typhimurium T3SS

**Figure 1:**
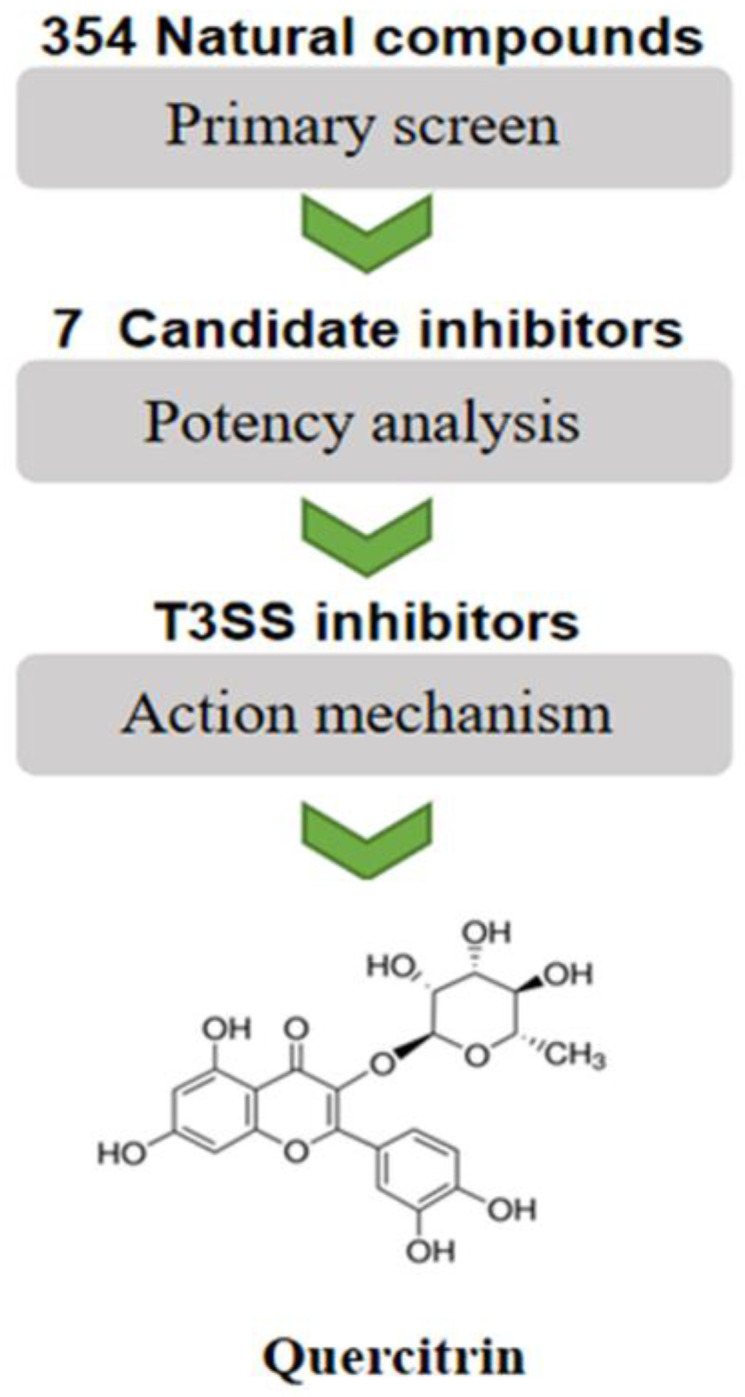
Flowchart for high-throughput screening of *S. Typhimurium* T3SS inhibitors.

According to a previous method [12], seven candidate compounds were preliminarily screened from 354 natural compounds established in our lab by using the SipA-β-lactamase fusion reporting system in *S. Typhimurium* (Figure 1). Through quantitative data analysis, it was found that only the response ratio of YL33 and quercitrin were less than 1.0, which could significantly inhibit the translocation of SipA to host cells (see Figure S1A,B in the Supplemental Material). Next, the LDH release rate of YL33 and quercitrin after co-culture with HeLa cells for 6 h was detected, and the compound YL33 had a negative effect on cell viability at low concentration (Figure S1C).

### 2.2. Quercitrin Blocks the Translocation of T3SS Effector SipA

**Figure 2:**
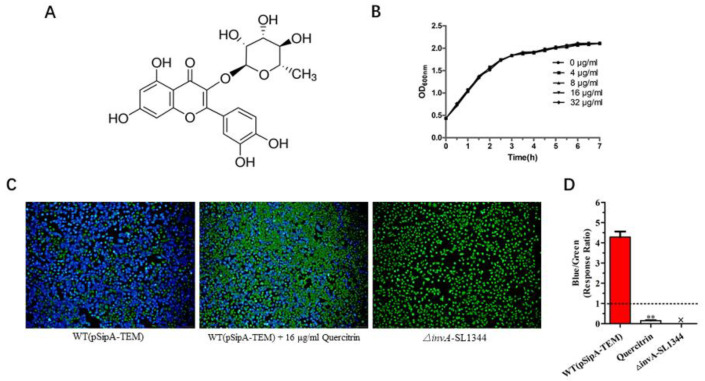
Quercitrin blocks the effector SipA translocation of T3SS. (**A**) Chemical structure of quercitrin. (**B**) Growth curve of *S. Typhimurium* SL1344 with quercitrin treatment for 6 h. (**C**) Quercitrin inhibits the effector SipA translocation to eukaryotic host cells of *S. Typhimurium* T3SS (Magnification 200×). The untreated group was set as 100%. WT (pSipA-TEM), positive control group. △*invA*-SL1344, negative control group. WT (pSipA-TEM) + 16 μL/mL Quercitrin, quercitrin treatment group. Blue fluorescence represents normal effector translocation, and green fluorescence represents blocked translocation. (**D**) Quantitative data of quercitrin inhibiting the effector SipA translocation were collected for statistical analysis by blue/green ratio (=response ratio) from five different visual fields. Blue/green ratio = blue cells/green cells. The response ratio meeting the baseline requirements should be at least less than 1.0, ** *p* < 0.01, compared with the WT (pSipA-TEM) group.

Quercitrin (Figure 2A) was selected for follow-up study due to it being the best inhibitor effect against T3SS activity. As shown in Figure 2B, quercitrin did not inhibit the activity of bacteria in the concentration of 4~32 μg/mL; it was preliminarily verified that the action mechanism was indirect sterilization or bacteriostasis. Fluorescence staining showed that quercitrin inhibited the translocation of T3SS effector SipA to eukaryotic host cells compared with the control group (Figure 2C), and the response ratio was 0.15 ± 0.05 at the concentration of 16 μg/mL, while the WT group was 4.29 ± 0.60 (Figure 2D).

### 2.3. Quercitrin Inhibits S. Typhimurium Adhesion in a Dose-Dependent Manner

**Figure 3:**
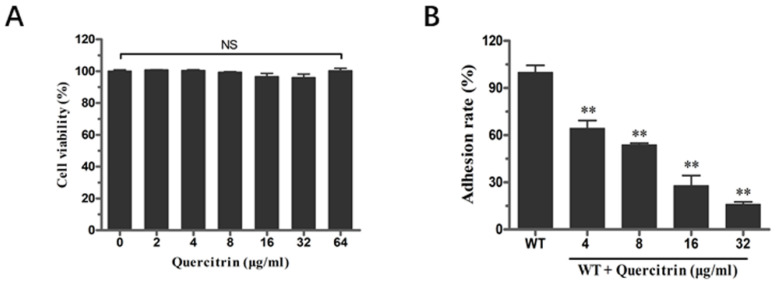
Quercitrin inhibits the adhesion of *S. Typhimurium* into HeLa cells without cytotoxicity. (**A**) Cytotoxicity of quercitrin on HeLa cells at different concentrations. (**B**) Inhibitory effect of quercitrin on adhesion of *S. Typhimurium* HeLa cells. ** *p* < 0.01 compared to the control group. NS, *p*  >  0.05, not significant.

The inhibition ability of quercitrin to inhibit *S. Typhimurium* adhesion to eukaryotic host cells was conducted according to the previous method [13]. Before the adhesion assay, the cytotoxicity of quercitrin to HeLa cells was determined by the lactate dehydrogenase (LDH) release assay. HeLa cells were co-incubated with different concentrations of quercitrin; the results showed that no cytotoxicity was observed in the concentration range of 2~64 μg/mL (Figure 3A). Next, bacteria cultured in the presence of quercitrin showed a significant reduction in the adhesion of HeLa cells in a dose-dependent manner. The adhesion effect of quercitrin was reduced to 15% compared to the untreated group at a concentration of 32 μg/mL (Figure 3B).

### 2.4. Quercitrin Inhibits S. Typhimurium Invasion in a Dose-Dependent Manner

**Figure 4:**
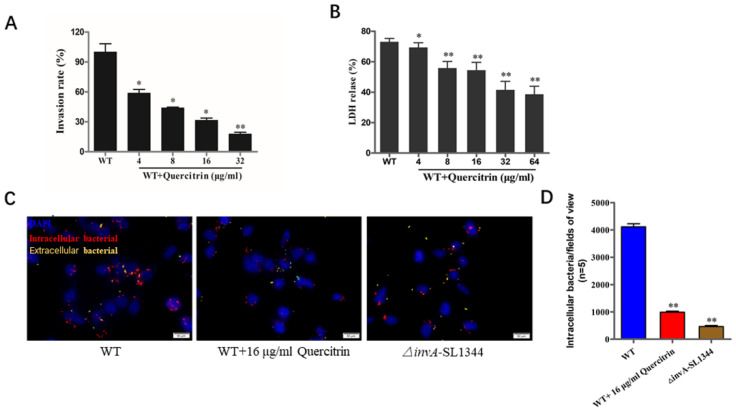
Quercitrin inhibits the invasion of *S. Typhimurium* into host cells and alleviates bacterial- mediated cell damage. (**A**) Inhibitory effect of quercitrin on the invasion of *S. Typhimurium*. HeLa cells were infected with *S. Typhimurium* SL1344 pretreated with quercitrin. Invasion rate = Intracellular bacteria of the treatment group/Intracellular bacteria of the WT group × 100%. The WT group without treatment was stated as 100%. (**B**) Quercitrin protects HeLa cells from *S. Typhimurium*-mediated damage. The LDH released data showed that the degree of HeLa cells damage caused by *S. Typhimurium* was significantly reduced after pretreatment with quercitrin. (**C**) Quercitrin significantly inhibits the invasion of *S. Typhimurium* (Magnification 1000×). Red fluorescence, intracellular bacteria; yellow fluorescence, extracellular bacteria; blue fluorescence, nuclei. (**D**) The immunofluorescence staining images of five different visual fields were collected, and the number of all invasive bacteria was counted by Image J (https://imagej.net/software/ accessed on 26 May 2023) (NIH, Bethesda, MA, USA) and compared with the untreated group and compared to the WT control group. * *p* < 0.05, ** *p* < 0.01 compared to the WT control group.

As an intracellular bacterium, adhesion and invasion are prerequisites for bacteria-mediated eukaryotic host cell damage. The quercitrin reduced *S. Typhimurium*-mediated invasion more than 70% at the concentration of 32 μg/mL (Figure 4A, see Table S2 in the Supplemental Material). As the data shown in Figure 4B, the LDH release rate of the wild-type bacterial infection group was about 70%, and the LDH release rate of bacterial infection in the quercitrin treatment group significantly decreased in a dose-dependent manner. As expected, immunofluorescence staining further demonstrated that quercitrin inhibited *Salmonella* invasion into HeLa cells (Figure 4C). The quantitative data showed that quercitrin significantly inhibited the adhesion of T3SS-dependent *S. Typhimurium* to eukaryotic host cells (Figure 4D).

### 2.5. Quercitrin Inhibits Expression of Related Genes and Effectors in T3SS

**Figure 5:**
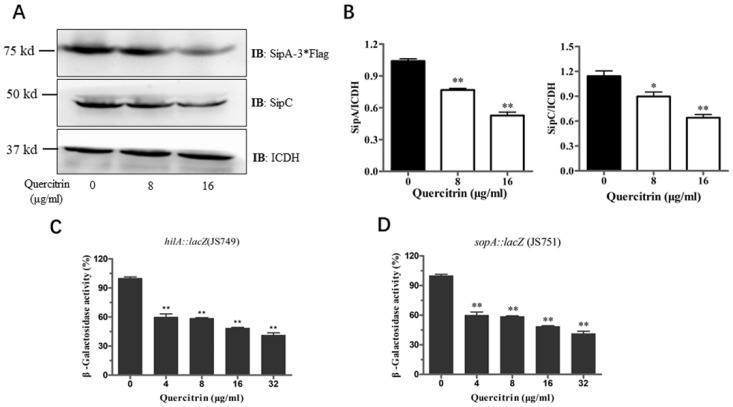
Quercitrin inhibits the expression of T3SS effectors and SPI-1 gene. (**A**) The effect of quercitrin on the expression of T3SS effectors SipA and SipC were detected by Western blot. (**B**) Quantitative analysis of the expression levels of effectors with Image J. (**C**,**D**) The activity of β-galactosidase was measured in cultures of *S. Typhimurium* expressing hilA::lacZ (JS749) and sopA::lacZ (JS751). * *p* < 0.05, ** *p* < 0.01 compared to the untreated group.

The above results showed that quercitrin inhibited the translocation of T3SS effector SipA. Western blot was used to detect whether quercitrin inhibited the expression of related effectors. It was found that quercitrin inhibited the expression of SipA and SipC in a dose-dependent manner (Figure 5A). Compared with the untreated group, quercitrin inhibited the expression of SipA by more than 50%, and the expression of SipC, another effector responsible for translocation and actin regulation, decreased by more than 30% (Figure 5B). β-Galactosidase activity assay was used to detect the expression of other important genes. The results showed that quercitrin significantly reduced the expression of *hilA* and *SopA*, which are mainly responsible for the regulation and function of *S. Typhimurium* T3SS (Figure 5C,D).

### 2.6. Quercitrin Improves Cecum Morphology and Reduces the Organ Colonization of Mice after S. Typhimurium Challenge

**Figure 6:**
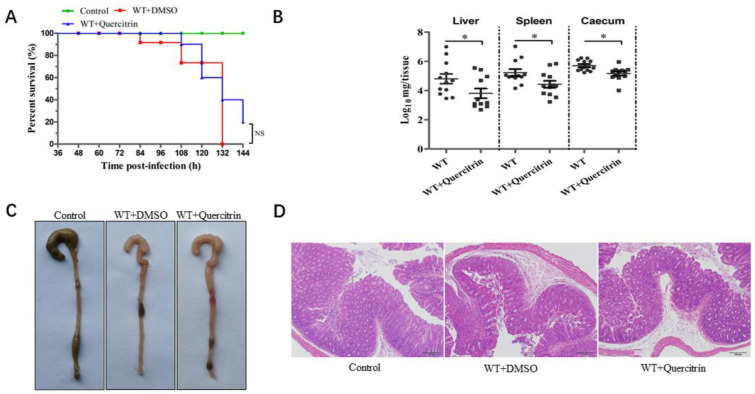
Quercitrin provides protection against *S. Typhimurium* infection in mice. (**A**) Quercitrin improves the survival rate of mice infected with *S. Typhimurium*. The survival rate was analyzed by Kaplan–Meier data statistics at 144 h. (**B**) Colony colonization in liver, spleen, and cecum at 96 h post-infection. (**C**) Observation of ocular lesions in cecum of control (without infection), WT + DMSO (model without treatment) and WT + quercitrin (quercitrin treatment group). (**D**) Histopathological changes of cecal tissue (scale is indicated “100 μm”). * *p* < 0.05 compared to the control group, NS, *p*  >  0.05, not significant.

The streptomycin-pretreated with *S. Typhimurium* infection mouse model was established according to previous research [14]. Mice were challenged with the *S. Typhimurium* SL1344 by oral gavage at a dose of 5 × 10^7^ CFUs, and the infection group was not treated. The results showed that the infection group began to die at 84 h, and the mortality was 100% within 132 h. In contrast, the quercitrin-treated group began to die at 108 h, and the survival rate was 20% within 144 h (Figure 6A). In other words, quercitrin delayed the beginning time of death for the infected mice by 24 h, and the survival rate of the infected mice increased by 20%.

At the same time, the colony colonization detection of target organs, ocular lesions of the cecum, and histopathological analysis of H&E staining were performed as previously described [15]. The data of colony colonization in target organs showed that, compared with the infected group, quercitrin significantly reduced the colony colonization in the liver, spleen, and cecum of the infected mice (Figure 6B). The ocular lesions of the cecum showed obvious characteristics of atrophy and intestinal wall thinning in the infection group, and there was no significant difference between the quercitrin treatment group and the control group (Figure 6C). The histopathological results showed typical characteristics such as submucosal edema, decreased proportion of goblet cells, epithelial cell abscission and necrosis, and polymorphonuclear cell (PMN) infiltration in lamina propria in the infection group, while quercitrin treatment significantly alleviated the pathological injury of the cecum (Figure 6D). In conclusion, quercitrin had a certain degree of protective effect against *S. Typhimurium* infection.

## 3. Discussion

Although new antibiotics, vaccines, and other strategies against pathogen infection are always in the development stage, the long development cycle and high economic costs prevent them from entering the market. At present, there are only a few mature preparations that are in clinical trials or nearing marketing. In recent years, antibiotic alternative strategies targeting pathogen virulence factors have shown promise. Since the virulence factors of many Gram-negative bacterial pathogens, rather than essential components for bacterial growth, depend on the multi-component type III secretory system injection structure (T3SSi), T3SSi has become an attractive target for identifying new drugs that inhibit its function to make the pathogen lose toxicity.

T3SS is widely distributed in Gram-negative bacteria, and its main function is to secrete and translocate pathogenic bacteria virulence factors [16]. Inhibition of *Salmonella* T3SS can achieve an anti- infection effect. Previous studies reported that inhibiting *Salmonella* T3SS could achieve an anti-infection effect and that significant progress has been made in the screening of inhibitors targeting the T3SS of *Salmonella*. For example, salicylidene acylhydrazides (SAHs) are the first identified and most widely studied class of synthetic small molecule inhibitors that target the T3SSi across many bacterial species [17]. Licoflavonol exerts a strong inhibitory effect on the secretion and expression of the *S. Typhimurium* SPI-1 effectors by regulating the transcription of the *SicA*/*InvF* genes and the translocation of the SipC [18]. Fortunately, quercitrin as a flavonoid could also target the type III secretion system to resist *Salmonella* infection, while the pharmacologic activities of quercitrin are similar to those of another flavonoid, licoflavonol. According to the research of Zhang et al., thymol could block the activity of T3SS-1 at a concentration that does not affect either the bacterial viability or the integrity of mammalian cell membranes [12]. Compared with the activity of thymol, which can reduce protein abundance that was related to the T3SS-1, quercitrin reduced the expression of SipA and SipC, which may indicate that quercitrin might interact with the Lon protein.

Although detection methods and screening models are constantly updated, the scarcity of small molecule inhibitors and the unclear action mechanism limits the discovery and clinical application of natural compounds or derivative inhibitors.

Compared with traditional antibiotics, most of these candidate inhibitors significantly reduce the possibility of inducing bacterial resistance by targeting the key virulence factors of the pathogen infection process, rather than relying on the action mechanism of bacteriostasis or sterilization [19]. However, it is important that the study of these molecules is helpful to clarify the structure–function relationship of the pathogen T3SS and serve as an efficient molecular platform for the development of active molecules targeting other T3SS homologous components [20].

Quercitrin belongs to the group of glycosylated flavonoids obtained from the whole grass of *Hypericum perforatum* and the bark of different species of oak trees with anti-bacterial, anti-inflammatory, and hypidemia effects [21]. In this study, quercitrin, a T3SS candidate inhibitor, was screened from 354 natural compounds using the SipA-TEM-β-lactamase fusion reporter system. The results showed that quercitrin inhibited the invasion of *S. Typhimurium* into host cells and protected the host cell from damage mediated by *S. Typhimurium* without affecting the growth of bacteria. Western blot and β-galactosidase activity assay showed that quercitrin significantly inhibited the expression of T3SS effectors SipA and SipC, and largely inhibited the expression of key effectors *hilA* and *sopA*. Limited by the extremely complex regulation network of *Salmonella* SPI-1 and the characteristics of other T3SSs, the current results only clarified the primary mechanism of quercitrin, which was also a limitation of this study and needs to be paid more attention in follow-up research. The results of the animal experiments showed that quercitrin could significantly reduce the colonization of target organ colonies, alleviated cecum pathological injury in mice infected with *S. Typhimurium*, and only improved the survival time and survival rate of infected mice to a certain extent. This may be related to the poor oral bioavailability and drug dosage form of quercitrin. In conclusion, quercitrin is an ideal lead compound for the treatment of *Salmonella* infection. The optimization and modification of its molecular structure and drug dosage form are solutions to improve its anti-infection effect. 

## 4. Materials and Methods

### 4.1. Bacterial Strains, Growth Conditions, and Natural Compounds

Professor Xiaoyun Liu of Peking University donated the wild-type *S. Typhimurium* SL1344. InvA is a prominent inner-membrane component of the T3SS apparatus, which is responsible for regulating effector export in pathogenic bacteria [22]; the *invA* mutant was used as a negative control in our study. The *hilA::lacZ* (JS749) and sopA::lacZ (JS751) strains were provided by Dr James Slauch from the University of Illinois. The strain that express 3×FLAG fused to SipA was constructed according to the research methods of Luo et al. [23]. The open reading frame of the gene was inserted into a pKS 3×Flag, which provides the 3×Flag prior to the insertion of fragments upstream and downstream of the fusion, respectively. This cassette was then inserted into the pir protein-dependent R6K vector pSR47S and the resulting plasmid was introduced into the SL1344 by triparental mating with the *E. coli* helper strain HB101 (pRK600). Transconjugants were streaked onto an LB plate containing 15% sucrose. Strains in which the tag was inserted properly into the chromosome were identified by diagnostic PCR with the appropriate primer sets (the primer sequences used are shown in Supplementary Materials Table S1). Bacteria were stored at −80 °C in Luria-Bertani (LB) broth containing 40% glycerol and overnight cultures grown in LB at 37 °C with aeration. All the natural compounds used, including the quercitrin, were preserved in our laboratory. Unless otherwise specified, they were all generally prepared at 40 mg/mL of dimethyl sulfoxide (DMSO) solution and stored at 4 °C.

### 4.2. High-Throughput Screening for T3SS Inhibitors Using SipA-β-lactamase Fusion Reporter System

HeLa cells were plated into 96-well plates (Clear, Round, Flat Bottom, Sterile) (Corning No.3359, USA) at a density of 1.2 × 10^4^ cells/well and incubated overnight before infection at 37 °C with 5% CO_2_. *S. Typhimurium* SL1344 containing SipA-β-lactamase fusion plasmid and the *invA* mutant strains was grown overnight at 37 °C in LB (0.3 M NaCl), and diluted 1:20 in the presence of quercitrin or DMSO control. After incubation for 4 h with shaking, the bacterial suspensions were adjusted to 3 × 10^6^ CFUs/mL. The HeLa cells were infected by 200 μL of the bacterial suspensions at a multiplicity of infection (MOI) of ~50 for 1 h. The non-internalized bacteria were washed three times with PBS and stained with 6×CCF4/AM reagent (Life Technologies, Frederick, MD, USA) for 45 min at room temperature. Fluorescence was examined by fluorescence microscopy (Olympus IX-81, Tokyo, Japan) and provided quantitative data (blue/green ratio = response ratio) including statistical analysis from three fields of view.

### 4.3. Determination of Bacterial Viability following Quercitrin Exposure

Bacterial viability following quercitrin exposure determination was performed by growth curve. The *S. Typhimurium* SL1344 cultures were diluted 1:100 in LB until the optical density of 600 nm (OD600nm) reached logarithmic growth phase. SL1344 were grown in the presence or absence of quercitrin. The OD_600nm_ were measured with the spectrophotometer (Unico UV-2100, Shanghai, China) every 30 min until the stationary phase.

### 4.4. Cytotoxicity of Quercitrin Indicated by Lactate Dehydrogenase Release

HeLa cells were inoculated into 96-well plates at a concentration of 1 × 10^5^ cells/mL. The adherent cells were subjected to 8 h of quercitrin exposure (at concentration of 0~64 μg/mL) in triplicate. DMEM and 0.2% Triton X-100 were set as the negative or positive controls. The release of lactate dehydrogenase (LDH) was measured using a LDH cytotoxicity detection kit (Roche, Penzberg, Germany) and microplate spectrophotometer (Tecan, Groding, Austria) at a wavelength of 490 nm [24]. After subtracting the absorbance of the DMEM control from the absorbance of each well, we calculated the LDH release rate of the cells according to the following formula: LDH release rate (%) = (absorbance of processed sample − absorbance of DMEM control hole)/(absorbance of TritonX-100 control − absorbance of DMEM control hole) × 100%.

### 4.5. Adherence Assay

In brief, the *S. Typhimurium* SL1344 pretreated with quercitrin at different concentrations for 4 h infected HeLa cells at an MOI of ~50. After incubation at 37 °C for 20 min, the 24-well plates were washed three times to remove unattached bacteria. The cells were lysed with 0.2% (*v*/*v*) Triton X-100 for 10 min and plated on LB agar plates for counting.

### 4.6. Gentamicin Protection Assay and Immunofluorescence Staining

The inhibition of quercitrin on bacterial invasion was determined by gentamicin protection assay and immunofluorescence staining with some modifications as described previously [25]. HeLa cells were plated in 24-well plates (4 × 10^4^ cells/well), *S. Typhimurium* SL1344 cultures were grown in fresh LB containing different concentrations of quercitrin for 4 h, and cells were infected by MOI of ~100 for 1 h. After washing three times with PBS to remove unattached bacteria, and being incubated in DMEM containing 100 µg/mL of gentamicin at for 50 min, the cells were lysed in 0.2% (*v*/*v*) Triton X-100 to enumerate the viable intracellular bacteria through a series of dilutions on LB agar plates. 

HeLa cells on coverslips were fixed with 4% paraformaldehyde and blocked with 5% BSA. Then, the monolayers were incubated with a primary antibody against *S. Typhimurium* (1:1000) overnight at 4 °C. The cells were subsequently incubated with the appropriate secondary Alexa Fluor 488-conjugated antibody (Abcam, Cambridge, MA, USA) for 30 min. Then, the cells were permeabilized with 0.3% (*v*/*v*) Triton X-100 for 10 min and subsequently incubated with the appropriate secondary Alexa Fluor 594-conjugated antibody (Abcam, USA) for 30 min. Finally, the cells’ nuclei were stained with 10 µL DAPI (Abcam, USA) for 10 min. Fluorescence was examined by fluorescence microscopy (Olympus IX-81, Tokyo, Japan).

### 4.7. Antibodies and Western Blot

Briefly, the SL1344, SipA-3×Flag-SL1344, and SipB-3×Flag-SL1344 cultures were added to fresh LB containing different concentrations of quercitrin. Each group was centrifuged at 12,000 rpm for 10 min (ThermoFisher scientific, Model:75002416, Waltham, MA, USA) and the precipitates were collected. We resuspended the bacteria in 100 μL using SDS-PAGE sample loading buffer (Tiangen RT209, Beijing, China) and boiled them at 95 °C for 6 min. Protein samples were separated using sodium dodecyl sulfate polyacrylamide gel electrophoresis (SDS-PAGE) and transferred to polyvinylidene difluoride (PVDF) membranes (Bio-Rad, Hercules, CA, USA).

The antibodies and dilutions were as follows: anti-SipC rabbit polyclonal antibody (1:2000, laboratory preservation), anti-Flag mouse monoclonal antibody (1:2000, CW Biotech, Beijing, China), anti-rabbit HRP or goat anti-mouse conjugated secondary antibodies (1:2000, Proteintech, Chicago, IL, USA), and anti-isocitrate dehydrogenase rabbit polyclonal antibody (ICDH, 1:2000) from Pro. Zhaoqing Luo of Purdue University served as an internal control. The immunoreactive protein bands were detected by using Enhanced Chemiluminescence ECL solution (Meilunbio MA0186-2, Dalian, China) and provided the quantitative data including statistics by Image J.

### 4.8. β-Galactosidase Activity Assay

According to a previously described method (26), overnight cultures of JS749 and JS751 strains were added to fresh LB in the presence or absence of quercitrin. We centrifuged the bacteria culture plates at 12,000 rpm for 10 min and resuspended them in Z-buffer. We then added 20 μL 0.1% SDS and 40 μL chloroform to the culture plates, shook and lysed for 30 s. 

We added 100 μL mixture into a new 96-well plate with three replicates of each group. We added 20 μL ONPG to initiate the reaction, incubated at room temperature for 10 min, and then added 50 μL 1 M Na_2_CO_3_ to terminate the reaction. The absorbance was detected at a wavelength of 450 nm using the microplate reader (Tecan, Groding, Austria).

### 4.9. Animal Experiment

All the experimental protocols were reviewed and approved by the Animal Welfare and Research Ethics Committee at Jilin University (SY202306059). The experiments were performed in strict compliance with the guidelines of the Animal Welfare Council of China. All efforts were made to minimize the suffering of the animals, and daily health checks were performed throughout the experiments. All the procedures were carried out in accordance with ARRIVE guidelines.

The animal study was conducted as previously described [26], 6~8-week-old female Balb/c mice were purchased from Changsheng BioTechnology (Shenyang, China). Three days before the experiment, 5 g/L of streptomycin was added to drinking water to interfere with intestinal flora, and the infection model was established by oral administration. Then, 30 mice were randomly divided into three treatment groups. Each group was given different treatments as follows: (1) oral administration and 50 mg/mL quercitrin treatment group, (2) the uninfected PBS control group, and (3) the infection without treatment group. Briefly, after fasting and banning water for 8 h before oral administration with 5 × 10^7^ CFU of SL1344, the quercitrin treatment group was given 50 mg/kg bodyweight subcutaneously at an interval of 12 h for 4 days. The survival rate within 144 h in each group was counted. Other evaluation indexes were carried out according to the following methods: the mice were infected by oral administration with 1 × 10^7^ CFU of SL1344, the quercitrin treatment group was given 50 mg/kg bodyweight at an interval of 12 h for 4 days, and then the mice in each group were killed by cervical dislocation after 96 h. The liver, spleen, and cecum tissues were removed by aseptic method. After weighing the organs, the tissue homogenates were prepared with PBS containing 0.2% Triton X-100, diluted, and inoculated on LB agar plates containing streptomycin. We counted the colonies after overnight culture at 37 °C. At the same time, the ocular lesions of the cecum were evaluated, hematoxylin and eosin (H&E) tissue sections were prepared, and the histopathological changes were observed by Optical Microscope.

### 4.10. Statistics Analysis

All experiments were conducted with least three biological replicates for analysis and statistical significance by GraphPad Prism 6.0 software (La Jolla, CA, USA). Results from the treated and control samples were expressed as the mean ± SEM and analyzed using Student’s *t* tests (* *p* < 0.05; ** *p* < 0.01; NS, *p* > 0.05, not significant).

## Data Availability

The data that support the findings of this study are available on request from the corresponding author. The data are not publicly available due to privacy or ethical restrictions.

## Supplementary Materials

The following supporting information can be downloaded at: https://www.mdpi.com/article/10.3390/molecules28145455/s1, Figure S1: Candidate compounds screened by SipA-β-lactamase fusion reporting system and pharmacological activity analysis; Table S1: The primers used in this experiment; Table S2: The absolute number of intracellular bacteria.
