# Supplementary material for: Quercitrin Is a Novel Inhibitor of Salmonella enterica Serovar Typhimurium Type III Secretion System
Source: Molecules. 2023 Jul 17;28(14):5455. doi: 10.3390/molecules28145455 (PMC10383848; doi:10.3390/molecules28145455)

Supplementary Material

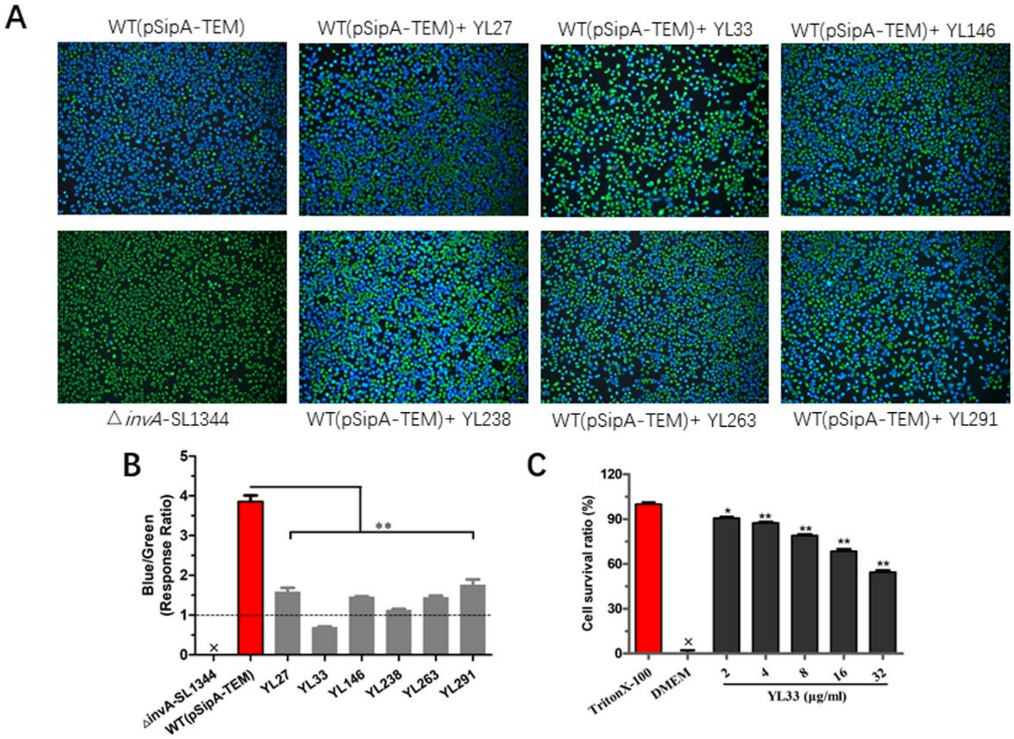

FIG S1 Candidate compounds screened by SipA-β-lactamase fusion reporting system and pharmacological activity analysis. (A) Fluorescence images of six compounds screened. (B) Quantitative data of six compounds inhibiting the translocation of effector SipA were collected for statistical analysis of blue/green ratio (=response ratio) from five different visual fields. The response ratio should be at least less than 1.0, meeting the baseline requirements. (C) Cytotoxicity of YL-33 on HeLa cells at different concentrations. **p* < 0.05; ***p* < 0.01 compared to the control group; ×, △invA-SL1344 group was not counted.

Table S1 The primers used in this experiment.

Primer name	Sequence (Restriction enzyme sites are underlined)	Note
SL1101	GTCGAGCTCGTGGTCACGTCAGAAAAGGGCA	*sipA* up Sac I Knock-in 3*flag
SL1102	TCCCCCGGGACGCTGCATGTGCAAGCCATCAACG	*sipA* up Sma I Knock-in 3*flag
SL1103	CGCCTCGAGTAATTAACCGGGAAAGATGCGATGA	*sipA* down Sac I Knock-in 3*flag
SL1104	CATGGTACCGTTCACCATTAATCACCATAA	*sipA* down Sma I Knock-in 3*flag
SL1105	CTTGTCGACGTTCACCATTAATCACCATAA	*sipA* down Sal I Knock-in 3*flag

Table S2 The absolute number of intracellular bacteria.

Repeats	WT (CFUs)	WT+ Quercitrin (μg/ml)			
		4 (CFUs)	8 (CFUs)	16 (CFUs)	32 (CFUs)
1	243000	115000	92000	75000	44000
2	199000	138000	89000	59000	36000
3	185000	116000	95000	64000	32000

Gels and blots images of Figure 5A

(1) SipA-3×Flag

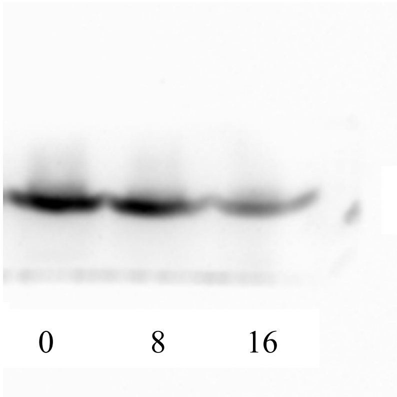

(2) SipC

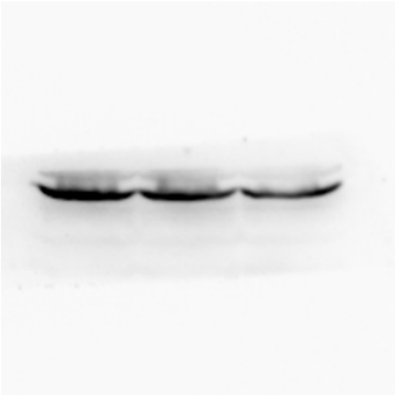

(3) ICDH

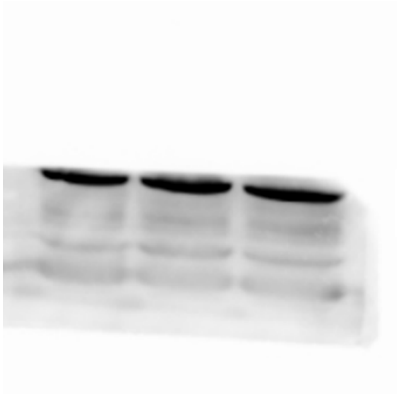

Supplement: Supplementary file 1 [file molecules-28-05455-s001.zip › molecules-2446841-supplementary.pdf]
